# Arousal, valence, and the uncanny valley: psychophysiological and self-report findings

**DOI:** 10.3389/fpsyg.2015.00981

**Published:** 2015-07-15

**Authors:** Marcus Cheetham, Lingdan Wu, Paul Pauli, Lutz Jancke

**Affiliations:** ^1^Department of Neuropsychology, University of ZurichZurich, Switzerland; ^2^Department of Psychology, Nungin UniversitySeoul, South Korea; ^3^Swiss Centre for Affective Sciences, University of GenevaGeneva, Switzerland; ^4^Department of Psychology, University of WurzburgWurzburg, Germany

**Keywords:** valence, arousal, uncanny valley hypothesis, familiarity, EMG, EEG, LPP

## Abstract

The main prediction of the *Uncanny Valley Hypothesis* (*UVH*) is that observation of humanlike characters that are difficult to distinguish from the human counterpart will evoke a state of negative affect. Well-established electrophysiological [*late positive potential* (*LPP*) and facial *electromyography* (EMG)] and self-report [*Self-Assessment Manikin (SAM*)] indices of valence and arousal, i.e., the primary orthogonal dimensions of affective experience, were used to test this prediction by examining affective experience in response to categorically ambiguous compared with unambiguous avatar and human faces (*N* = 30). LPP and EMG provided direct psychophysiological indices of affective state during passive observation and the SAM provided self-reported indices of affective state during explicit cognitive evaluation of static facial stimuli. The faces were drawn from well-controlled morph continua representing the UVH’ *dimension of human likeness* (*DHL*). The results provide no support for the notion that category ambiguity along the DHL is specifically associated with enhanced experience of negative affect. On the contrary, the LPP and SAM-based measures of arousal and valence indicated a general increase in negative affective state (i.e., enhanced arousal and negative valence) with greater morph distance from the human end of the DHL. A second sample (*N* = 30) produced the same finding, using an *ad hoc* self-rating scale of feelings of familiarity, i.e., an oft-used measure of affective experience along *the UVH’ familiarity* dimension. In conclusion, this multi-method approach using well-validated psychophysiological and self-rating indices of arousal and valence rejects – for passive observation and for explicit affective evaluation of static faces – the main prediction of the UVH.

## Introduction

**FIGURE 1:**
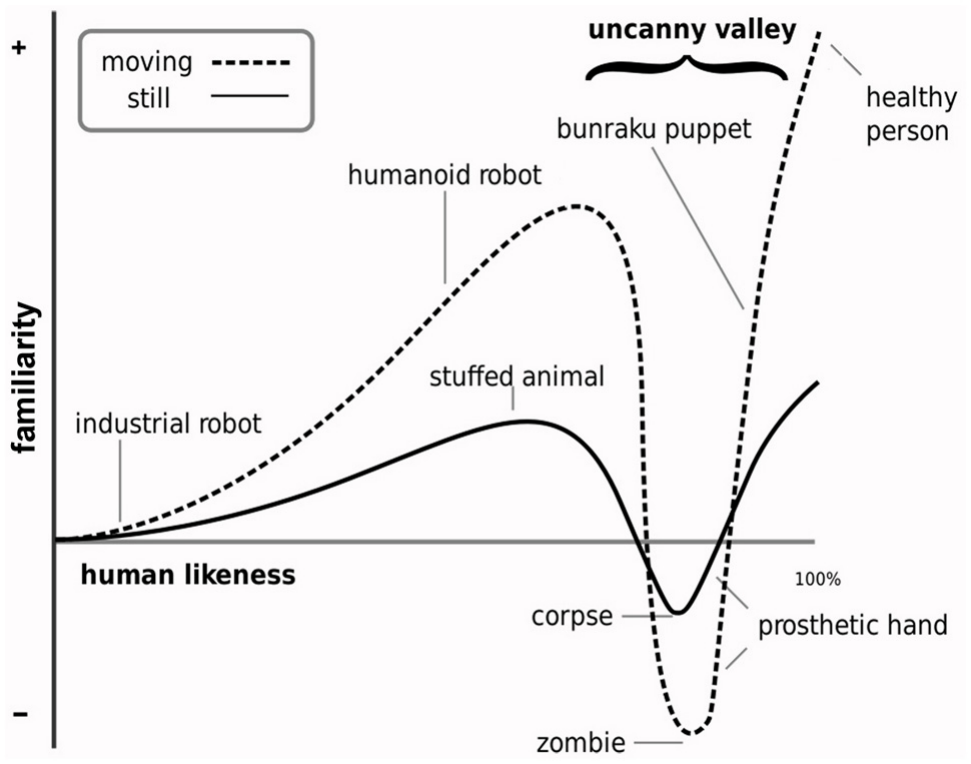
**Illustration of the Uncanny Valley Hypothesis.** The main prediction of the Uncanny Valley Hypothesis is that observation of highly humanlike characters and objects (depicted along the dimension of human likeness) will evoke a sharp negative peak in affective experience (depicted along the familiarity dimension). This negative peak, referred to as the uncanny valley, is characterized by feelings of strangeness and disquiet. These feelings are suggested to be stronger for dynamic stimuli. The valley occurs at the point along the dimension of human likeness at which category membership as human or non-human is highly ambiguous (illustration adapted from MacDorman, 2005).

The longstanding *Uncanny Valley Hypothesis* (*UVH)* predicts that difficulty distinguishing a realistic humanlike character or object (e.g., robot, prosthetic hand) from its human counterpart will evoke an unpleasant affective state (Mori, 1970; **Figure 1**). Mori suggests that this state is characterized by a sense of strangeness and personal disquiet and, when experienced more intensely, by revulsion and disgust. Attention to the originally untested UVH has been spurred by recent progress in robotics and computer graphics technologies in the realistic simulation of aspects of human appearance and behavior and, therefore, by interest in understanding the impact of enhanced anthropomorphic realism on affective experience (e.g., Ho and MacDorman, 2010; Yamada et al., 2013). But empirical support for the predicted uncanny effect has been inconsistent (e.g., Hanson, 2006; MacDorman, 2006; Tinwell and Grimshaw, 2009; MacDorman et al., 2013). This has led to the query as to how research should now best proceed (Zlotowski et al., 2013). To move beyond this seeming impasse, some of the reasons for inconsistency in findings and new avenues of approach can be considered.

One reason for inconsistency stems from the UVH’ ambiguous definition of the concept *shinwakan*. Mori coined this Japanese neologism to describe in simple terms the positive and negative character of affective experience in response to variously humanlike objects (**Figure 1**); the UVH defines these objects along a *dimension of human likeness* (*DHL*). Though relating to affective experience, the poor specification of this concept has resulted in much debate and various renderings of its meaning, with the investigation of the UVH and shinwakan in terms of constructs such as pleasantness, comfort level, eeriness, familiarity (i.e., feelings of familiarity vs. strangeness), likability, and empathy (e.g., MacDorman and Ishiguro, 2006; Bartneck et al., 2007; Seyama and Nagayama, 2007, 2009; Green et al., 2008; MacDorman et al., 2009a, 2013; Tinwell and Grimshaw, 2009; Dill et al., 2012; Burleigh et al., 2013). Adding to, or reflecting, the UVH’ conceptual ambiguity, Mori (2012) recently re-termed the positive and negative character of affective experience as affinity.

An alternative approach to investigating affective experience of human and humanlike entities is to consider affective experience in terms of its psychologically well-validated components. Mori (1970) uses illustrative instances of feelings and affective evaluations to describe his understanding of shinwakan. Based on these instances, shinwakan could well be considered in terms of the constructs *valence* and *arousal* because these are intrinsic to all of his examples (cf. Davitz, 1969). These are also intrinsic to the affective dimensions (e.g., likeability, pleasantness, feelings of familiarity) typically used to examine the UVH and to all of the early theoretical accounts of uncanny experience (e.g., MacDorman, 2005), and they appear to be relevant for the definition of uncanny feelings in terms of specific emotions (cf. Russell, 1980; Ho et al., 2008). Valence refers to the pleasant-to-unpleasant quality (hedonic tone) and arousal to the low-to-high degree of excitement of affective experience (Barrett and Russell, 1999; Kensinger and Corkin, 2004). Given that valence and arousal form the primary orthogonal dimensions of affective experience (Schlossberg, 1954; Russell, 1980, 2003; Yik et al., 1999; Posner et al., 2005), that emotional states can be defined in the emotional space determined by these two dimensions (Bradley and Lang, 1994; Barrett and Russell, 1999), and that the principal variance in the meaning of emotional states can be explained by valence and arousal (Osgood et al., 1957; Mehrabian and Russell, 1974; Smith and Ellsworth, 1985), it is likely that indicators of valence and arousal will provide a sound basis for examining affective experience of variously humanlike entities along the DHL and the notion of the uncanny effect.

A second reason for inconsistent findings is likely to relate to the more or less exclusive reliance in uncanny-related research on *ad hoc* developed self-rating scales to assess affective experience (e.g., Hanson, 2006; MacDorman, 2006; Green et al., 2008; MacDorman et al., 2009a,b; Seyama and Nagayama, 2009; Looser and Wheatley, 2010; Tinwell et al., 2010). *Ad hoc* self-rating scales have found favor in uncanny research because they are inexpensive and easy to administer (see, e.g., Ho et al., 2008). But the psychometric validity and reliability of these measures as indicators of *shinwakan*, or of the affective experience that the notion of *shinwakan* is thought to capture, has not been demonstrated (but for steps toward construct validation, see Ho and MacDorman, 2010). This makes the interpretation and synthesis of the research findings to date difficult.

As an alternative to *ad hoc* scales, and in keeping with the foregoing considerations on arousal and valence, well-validated measures such as the *Self-Assessment Manikin* (*SAM*, e.g., Lang, 1985; Lang et al., 1997; Bradley and Lang, 2007) could be used. The SAM is a pictorial assessment technique for self-rated measurement of valence and arousal. As a non-verbal and largely culture free measure (Morris et al., 1993; Bradley et al., 1994), it would be useful – given the preceding concerns about the ambiguous concept shinwakan – for application in uncanny-related research. Though a valuable source of information, self-ratings of affective experience (e.g., of pleasantness, comfort level, eeriness, familiarity, likability, valence, or arousal) effectively place the focus of investigation on conscious feelings of emotional state (Barrett, 1996), that is, on the explicit cognitive evaluation of the affect-related properties of the stimuli and of the psychophysiological reactions that these stimuli elicit. It is, however, conceivable that the proposed uncanny effect might also manifest itself in affect-related reactions that escape conscious detection and evaluation (for affect-related neural processes during passive category processing of faces along the DHL, see Cheetham et al., 2011). Self-report measures can be augmented therefore by direct measures of psychophysiological reactions. For the present study, we considered the use of *facial electromyography* (*EMG*) and *electroencephalography* (*EEG*) to indicate rapid and subliminal changes in emotional state (Wu et al., 2012). Affective experience is associated with psychophysiological indices that correlate differentially with valence and arousal (Cacioppo et al., 1986; Bradley and Vrana, 1993; Lang et al., 1993, 1997; Schupp et al., 2000; Amrhein et al., 2004; Foti and Hajcak, 2009): the EMG measures of the corrugator supercilii muscle and the zygomaticus major muscle are sensitive to negative and positive valence, respectively, and the EEG-based measure of the *late positive potential* (*LPP*) to arousal (Cacioppo et al., 1986; Lang et al., 1993, 1997; Cuthbert et al., 2000; Schupp et al., 2000; Dolcos and Cabeza, 2002; Amrhein et al., 2004; Hajcak and Nieuwenhuis, 2006; Foti and Hajcak, 2009; Hajcak et al., 2009; Weinberg et al., 2012; Wu et al., 2012).

A further reason for inconsistency in findings has been suggested to relate to the conceptualization and operationalization of the DHL (Cheetham et al., 2011). Mori’s illustration of the UVH uses a single human exemplar to represent the human category (Mori, 1970), as reflected in some empirical and theoretical work (e.g., Ramey, 2005; Tinwell and Grimshaw, 2009). But this conceptualization effectively assumes that there is no variation in physical or psychological similarity space within the human category of the DHL. Examination of perceptual discriminative and category processing along the DHL, operationalized using morph continua to represent a linear dimension of physical similarity space spanning between human and non-human category exemplars, shows that this assumption is incorrect (Cheetham et al., 2011, 2014). The use of linear morph continua to represent the DHL is not new (e.g., Seyama and Nagayama, 2007), but poor control of continua might have contributed to inconsistency in findings. Morph continua have been subject to various experimental confounds that are likely to have systematically biased subjective experience of objects along the DHL (for a critical discussion, see Cheetham and Jancke, 2013). These confounds range from the use of different juxtaposed morph continua to represent the DHL, thus generating perceptual discontinuities along the morph continua (Hanson et al., 2005; MacDorman and Ishiguro, 2006) to morphing noise (e.g., disparities in the alignment of facial features between successive morphs of a continuum), as indicated in a recent study of the effects on subjective experience of category ambiguity (Yamada et al., 2013). Critically, morphing noise is likely to alter the cognitive representation of the human–nonhuman category structure of the DHL and may itself influence subjective experience (Cheetham and Jancke, 2013).

Understanding the human–nonhuman category structure of the DHL provides an approach to testing the ideas underlying the vaguely formulated UVH (Cheetham et al., 2011, 2014; Burleigh et al., 2013; Yamada et al., 2013; Burleigh and Schoenherr, 2015; Ferrey et al., 2015). The UVH makes no explicit reference to perceptual and category processing or to the large body of pertinent literature. But, based on this understanding, Mori’s ideas can be tested by augmenting the UVH with the assumption that the predicted state of negatively valenced affect is most likely to occur at the point of realism along the DHL at which attribution of stimuli to the human or non-human category is subject to greatest categorization ambiguity (i.e., the “valley” in **Figure 1**). A similar approach has been applied to examining the relationship between the predicted state of negatively valenced affect and the ability to perceptually discriminate between (rather than categorize) morphs along continua representing the DHL (Cheetham et al., 2014). Cheetham et al. (2014) reported a number of effects that, however, provided no support for Mori’s ideas.

The aim of this study was to examine affective experience in response to the presentation of highly similar human and humanlike facial stimuli along the DHL. In view of the uncertainty surrounding the conceptual definition and translation of shinwakan, we focused in the main experiment (i.e., the first of two experiments) on the examination of valence and arousal as two of the primary properties of affective experience, using non-verbal measures. The facial *EMG* measure of the corrugator supercilii muscle and the LPP were used as psychophysiological indices of valence and arousal, respectively. The SAM was used to assess self-reported valence and arousal. The DHL was represented using morphs drawn from carefully controlled continua generated from avatar and human faces. These continua were previously tested to ensure that the cognitive representation of the category structure (i.e., morph location of categorically highly unambiguous avatar and human face exemplars and of categorically most ambiguous faces) was consistent across continua.

To test the main prediction of the UVH, we assumed that the face morph associated with greatest category ambiguity along the DHL would evoke greater negatively valenced and more arousing affective experience compared with that evoked by categorically unambiguous avatar and human morphs. In the second experiment, we examined the relationship between the DHL and shinwakan in order to provide, using our stimuli, a general reference of comparison with previous studies that have focused on shinwakan using *ad hoc* scales. For this, an *ad hoc* self-rating scale of shinwakan based on the bi-polar dimension of *familiarity* (i.e., feelings of familiarity vs. strangeness) was used. Familiarity was selected because this rendering of shinwakan has been frequently investigated and because it closely captures the essence of Mori’s description of the uncanny. In keeping with the preceding considerations on category structure, and assuming for the purpose of experimentation that Mori’s conjectures are correct, self-rated experience of familiarity was expected to show that feelings of strangeness would be greater along the DHL for categorically ambiguous morphs compared with categorically unambiguous avatar and human morphs.

## Materials and Methods

### Participants

Healthy male and female adults with no record of neurological or psychiatric illness and no current medication use volunteered for one of the two studies. Participants in the first experiment, conducted in Wurzburg, were students of the University of Wurzburg and those of the second experiment, conducted in Zurich, were students of the University of Zurich. All participants were native or fluent speakers of Standard German, consistently right-handed (Annett, 1970), and had no previous experience designing or modifying computer-generated characters in, for example, virtual reality-based role-playing games, second life, or virtual reality environments, or experience using such environments (e.g., for psychotherapy, rehabilitation, training, e-commerce, or virtual reality-based research). Written informed consent was obtained before participation according to the guidelines of the Declaration of Helsinki. Each volunteer received 20 Swiss Francs or the equivalent in Euros for participation. The study and all procedures and consent forms were approved by the Ethics Committee of the Universities of Wurzburg and Zurich.

### Materials and Stimuli

Twenty linear morph continua were generated, using FantaMorph software (Version 5.3.5, Abrosoft^1^), from 20 different pairs of color images of avatar and natural human faces. Each pair represented the two endpoints of a morph continuum, the continua being used to represent the DHL (for an example of stimuli used in this study, see Figure and Supplementary Figure S1B). Each continuum comprised 13 different morphed images, labeled M0 (avatar endpoint) to M12 (human endpoint), with each morph position representing an equally spaced-point along its respective continuum at increments of 8.33%. All faces were unknown and male, showing full face, frontal view, neutral expression, direct gaze, and no salient features such as facial hair and jewelery. The modeling suite Poser 7 (Smith Micro Software^2^) was used to generate and model in detail the facial geometry and texture (e.g., age, configural cues, skin tone) of the avatar faces to closely match the corresponding human face of the respective continua. The images were then edited in Adobe Photoshop CS3 to mask external features with an elliptic form and black background (96 dpi and 560 × 650 pixels), to ensure final alignment of avatars and human facial features, and to match contrast levels and overall brightness of each pair of parent faces before morphing.

The 20 continua (260 stimuli in total) were used to ensure a sufficient number of trials for signal averaging across continua in order to enhance the signal-to-noise ratio for the LPP and EMG measures. The final choice of continua was based on three pilot studies (*N* = 82). Two of these pilot studies used a *two-alternative forced choice classification task* to verify the consistency of the category structure of the DHL across the continua. This task required that the participants identify the presented stimulus as either an avatar or human as quickly and accurately as possible after stimulus onset by pressing one of two response keys. These pilots showed that faces at morph positions M0, M1, M2, and M3 were highly unambiguously assigned to the avatar category, faces at M9, M10, M11, and M12 were highly unambiguously assigned to the human category, and that M6 was most closely associated with greatest ambiguity in categorization judgments (for details and Supplementary Figure S1A, see Supplemental Information 1). Another pilot study (*N* = 18) was used to judge the facial attractiveness of avatar and human endpoints of all continua before these were morphed. A dependent sample *t*-test showed that there was no significant difference in attractiveness ratings between the avatar (*M* = 2.76, SD = 0.44) and human parent images (*M* = 2.87, SD = 0.52), *t*_17_ = -1.076, *p* = 0.297.

## Experiment 1: Psychophysiological Recordings and Ratings of Valence and Arousal

### Participants

Of *N* = 30 participants, three were excluded before data analyses because of excessive impedances during facial EMG and EEG acquisition, leaving *N* = 27 participants aged between 18 and 36 years (15 female; *M* = 23 years; SD = 4.02).

### Materials and Procedure

All participants were tested individually. Participants were seated in a small, sound-attenuated, dimly lit, shielded cabin, and electrodes for EMG and EEG acquisition were attached. Participants then provided demographic information and completed the *State Trait Anxiety Inventory* (*STAI*; Spielberger et al., 1970; German version by Laux et al., 1981). A 1 min resting baseline was performed at the beginning of the experiment to facilitate laboratory adaptation. Each participant received written instructions presented on the screen before commencement of each of three tasks, which were always performed in the same order (in keeping with standardized procedure, e.g., Amrhein et al., 2004).

Three tasks were conducted. Each task presented the same 260 stimuli (550 × 650 pixels) at a viewing distance of 62 cm and subtended a visual angle of 11° × 14°; this is approximately equivalent to viewing a real face from a normal distance during conversation of 90–100 cm (Hall, 1991; Henderson et al., 2005). The stimuli were always presented individually and in random order, with the constraint that no stimuli from within the same continuum or from corresponding morph positions of the different continua were shown in sequence.

In Task 1, participants viewed the stimuli for the duration of each stimulus’ presentation, without any further task requirement. Each trial began with a stimulus that timed out at 750 ms. followed by the *inter-trial interval* (*ITI*). The ITI varied randomly (between 4,000 and 5,000 ms), showing a black screen with a white fixation cross. EMG and EEG psychophysiological measures were recorded concomitantly. In Task 2, the stimuli were presented to the participants using a computerized version of the SAM. This required that participants press an appropriate key to indicate their subjective ratings of valence and arousal for each stimulus; the SAM rating scales range from 1 to 9 (i.e., very positive to very negative for valence and very high to very low for arousal). The button press was followed by the ITI (as in Task 1). A practice pre-test of five trials using stimuli from continua not included in the main test was performed to ensure correct use of the SAM rating scales. In Task 3, a *two-alternative forced choice classification task* was conducted to verify the location of the morph associated with greatest categorization ambiguity and the profile of avatar and human category decisions for the other morphs; please note that this task is the same as the two-alternative forced choice classification task used in the pilot studies. This task required that participants press an appropriate key to indicate their category judgments. The button press was followed by the ITI (as in Task 1), with time out at 750 ms. A practice pre-test of five trials using stimuli from continua not included in the main test was performed to ensure correct use of the response buttons and comprehension of the category label ‘avatar.’

### Psychophysiological Data Recording and Reduction

Continuous psychophysiological recording was performed in Task 1. EMG acquisition entailed bipolar placement of Ag/AgCl electrodes with surface diameter of 7 mm over the left M. corrugator supercilii (Fridlund and Cacioppo, 1986). Participants were told that skin conductance would be recorded (Dimberg et al., 2000; Weyers et al., 2006, 2009; Likowski et al., 2012). The EMG raw signal was measured with a V-Amp 16 amplifier (Brain Products Inc., Gilching, Germany) and stored with a sampling frequency of 1000 Hz. Raw data were then rectified and filtered oﬄine with a 30 Hz low pass and 500 Hz high pass cut-off filter, a 50 Hz notch filter, and integrated with a 125 ms time constant. The EMG difference scores were computed on the basis of the mean change in activity after stimulus onset from 500 ms baseline before stimulus onset. Trials with EMG activity exceeding 8 μV during the 500 ms baseline and above 30 μV during stimulus presentation were excluded (less than 5%). For statistical analyses, the data of each participant were collapsed over the 20 trials of each of the 13 corresponding morph positions of the 20 continua and averaged over the 100 ms intervals post-stimulus onset (Weyers et al., 2006; van Boxtel, 2010).

For the LPP, EEG was recorded using Ag/AgCl electrodes placed at mid-line sites according to the international 10–20 system (i.e., sites FCz, Cz, CPz, Pz, C1, C2, CP1, CP2) at a sampling rate of 1,000 Hz and referenced to Cz during data recording and replaced by the mean of mastoids during off-line data analysis. Electrodes were mounted on an Easycap (EasyCap, Hersching, Germany). Raw data were processed and analyzed using the computer Brain Vision Analyzer software (Version 2.0, Brain Products Inc.). The continuous EEG data were subjected to band-pass between 0.01 and 20 Hz filter off-line. Trials with EEG activity exceeding a transition threshold of 50 μVolt (sample to sample) or amplitude of 300 μV were excluded from further analysis. EEG data was corrected for blinks and eye movement artifacts (Gratton et al., 1983). Data for the LPP was extracted for stimulus synchronized epochs from 100 ms baseline preceding stimulus onset till 750 ms post-stimulus onset. The data was then baseline corrected (i.e., the 100 ms before stimulus onset), and then averaged for each participant and each of the 13 corresponding morph positions of the 20 continua. The LPPs were determined on the basis of mean amplitude calculated over time windows on the basis of the literature (e.g., Schupp et al., 2000). In particular, LPPs were scored as mean activity between 300 and 750 ms after stimulus onset over the midline electrode sites (CPz, CP1, and CP2; Schupp et al., 2004; Hajcak et al., 2007, 2010; Foti and Hajcak, 2008).

All data analyses in this and the following experiment were performed using SPSS version 18.0 (SPSS, Inc., Chicago, IL, USA).

## Results

### STAI Questionnaire

The average score of the STAI state scale was *M* = 36.07 (SD = 7.96, range = 22–55) and that of the STAI trait scale was *M* = 36.42 (SD = 10.19, range = 20–60); the STAI scales measure anxiety, ranging from 20 (not at all anxious) to 80 (very anxious).

### Categorization Responses

To verify the choice of categorically ambiguous and unambiguous morphs for further analyses, informal inspection of the results of the two-alternative forced choice classification task (Task 3 of Experiment 1), indicates that morph position M6 is associated with greatest ambiguity in avatar-versus-human categorization responses and that the avatar (i.e., M0, M1, M2, M3) and human faces (i.e., M9, M10, M11, M12) show a lower and upper asymptote that nears 95% (see Supplementary Figure S2 in Supplemental Information 2); this profile of category judgments is consistent with the pilot data (see Supplemental Information 1).

**FIGURE 2:**
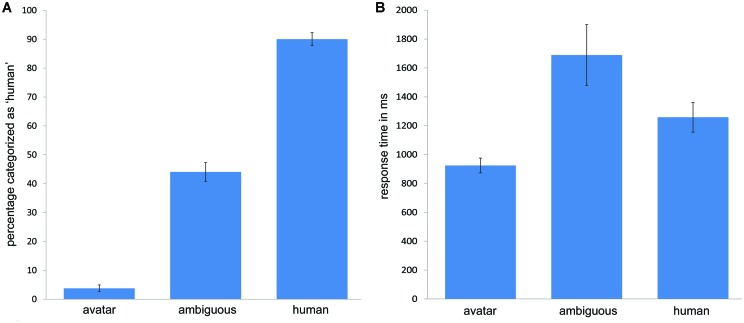
**Mean categorization responses and response times in the two-alternative forced choice categorization task.** Mean categorization responses are depicted in terms of percentage of ‘human’ responses **(A)** for morphs representing unambiguous *avatar* and *human* faces and the most *ambiguous* face along continua representing the DHL. The depiction of corresponding mean response times for categorization responses **(B)** show longer response latencies for ambiguous faces and the shortest response latencies for avatar faces. The error bars indicate standard errors (*N* = 27).

To characterize this profile more clearly, the mean categorization response data for M6 were compared with the aggregated mean data for the avatar faces and human faces. Greenhouse–Geisser adjustment was applied to correct the degrees of freedom for violation of the sphericity assumption as appropriate in this and subsequent analyses. A one-way *repeated measures of analysis of variance* (*RM-ANOVA*) with the factor *morph* position (three levels: M6, ‘M0, M1, M2, M3’ and ‘M9, M10, M11, M12’) was conducted on the dependent variable categorization *response* for each participant across continua. The categorization responses were entered in the analysis in terms of percentage of responses categorized as human. This analysis showed a highly significant effect for the expected differences for morph position in categorization responses, *F*(2,52) = 347.42, *p* < 0.001, with M6 approaching chance level of 50% in categorization responses (*M* = 0.44; SD = 0.17), whereas the morphs at M0, M1, M2, and M3 and at M9, M10, M11, and M12 were clearly judged to be exemplars of the avatar (*M* = 0.04; SD = 0.06) and human face categories (*M* = 0.9; SD = 0.12), respectively (see **Figure 2A**).

### Categorization Response Times (RTs)

The longest response latency might be expected to correspond with greatest categorization ambiguity along the DHL (Cheetham et al., 2011, 2013). To confirm this for the present data (though RT is not relevant for the Tasks 1 and 2 in Experiment 1), the same *RM-ANOVA* as in the preceding was applied using categorization *response times (RTs*; in ms) rather than categorization response as the dependent variable. Given the reported asymmetries in processing avatar and human faces along the DHL (Cheetham et al., 2011, 2013), this and the following analyses included pre-planned contrasts to compare measures for M6 and the human and avatar faces. This analysis showed a significant effect for morph position along the DHL, *F*(1.11,29.06) = 17.13, *p* < 0.001. The pre-planned contrasts showed a significant difference between RT at M6 (*M* = 1689; SD = 1091) compared with the mean average for avatar *and* human morphs (*M* = 1097; SD = 551), *F*(1,26) = 20.41, *p* < 0.001, indicating that RT is significantly longer at M6 than for other morphs. Consistent with previous RT data (Cheetham et al., 2011, 2013; Cheetham and Jancke, 2013), pre-planned contrasts showed a significant difference in RT between the avatar (*M* = 924; SD = 276) and human morphs (*M* = 1258; SD = 534), *F*(1,26) = 22.58, *p* < 0.001, such that the mean latency of categorization responses was longer for human faces (see **Figure 2B**).

### Affective Experience and Categorization Ambiguity

Based on the preceding, we compared the lpp, emg, sam for valence and sam for arousal data at m6 with the data for the avatar and human category faces. Separate one-way rm-anovas with the factor *morph* position (three levels: m6, ‘m0, m1, m2, m3’ and ‘m9, m10, m11, m12’) were conducted for each of the dependent variables lpp, emg, sam ratings for valence, and sam ratings for arousal of each participant across the 20 continua.

**FIGURE 3:**
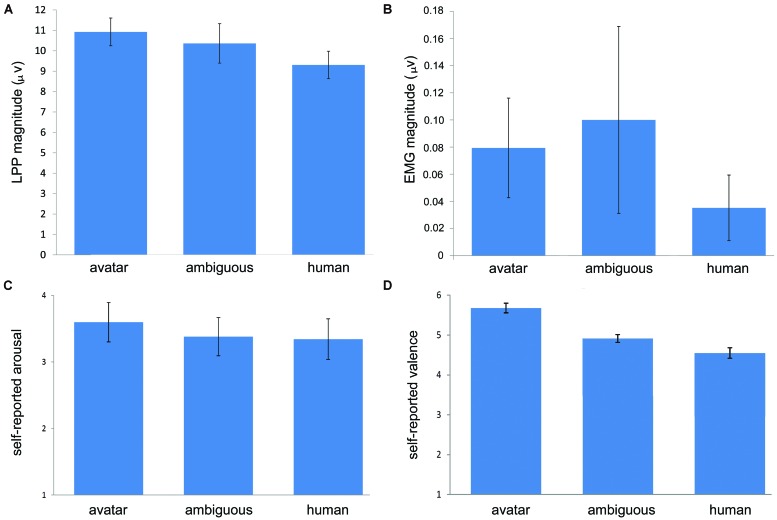
**Effect of highly unambiguously avatar and human and highly ambiguously categorized faces on arousal and valence.** Overall, the figures illustrate a general increase in LPP magnitude indicating arousal **(A)**, in self-rated arousal **(C)** and in self-rated negative valence **(D)** with decreasing human likeness from the human to the avatar faces. Differences in EMG magnitude indicating negative valence **(B)** are not significant. The error bars indicate standard errors (*N* = 27).

**FIGURE 4:**
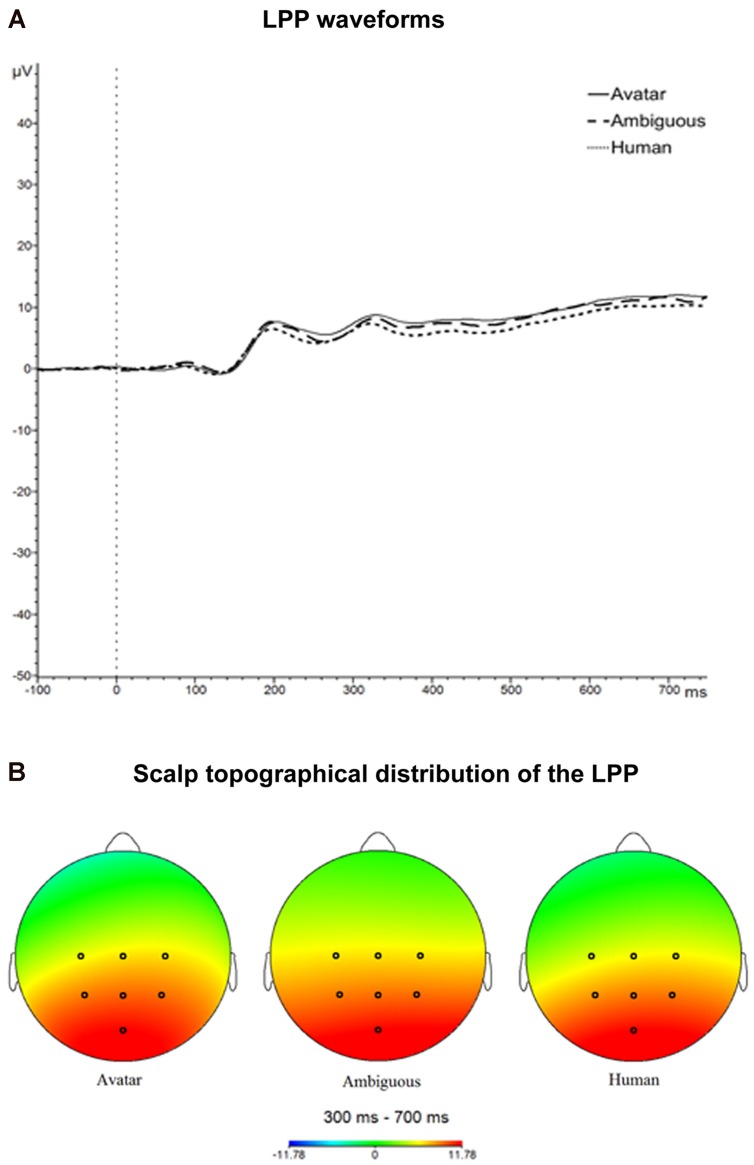
**Late positive potential (LPP) waveforms and scalp topographical distribution.** The figure illustrates the LPP waveforms **(A)** and the scalp topographical distribution of the LPP **(B)** for avatar faces, ambiguous faces and human faces.

For lpp, there was a significant effect of morph position along the DHL, *F*(1.31,34.09) = 5.62, *p* = 0.016. Pre-planned contrasts showed a significant difference between the avatar (*M* = 10.92; SD = 3.54) and human faces (*M* = 9.31; SD = 3.5), *F*(1,26) = 36.31, *p* < 0.001, such that the measure for LPP was greater for the avatar than for the human category (see **Figures 3A** and **4**). The pre-planned contrasts showed no significant difference between M6 and the avatar or the human faces, respectively. These data indicate that the LPP values increased across the three stimulus conditions (i.e., avatar faces, M6 and human faces) with increasing morph distance from the human end of the continua.

For EMG and the corrugator supercilii activity (see **Figure 3B**), there was no significant effect of morph position, *F*(1.38,35.83) = 0.66, *p* = 0.47.

There was a significant effect of morph position for the SAM arousal ratings, *F*(1.51,39.38) = 3.78, *p* = 0.043. Pre-planned contrasts showed a significant difference between the avatar morphs (*M* = 3.6; SD = 1.54) and M6 (*M* = 3.38; SD = 1.49), [*F*(1,26) = 4.43, *p* = 0.045] and between the avatar and the human morphs (*M* = 3.34; SD = 1.58), [*F*(1,26) = 4.45, *p* = 0.045], such that arousal ratings increased across the three stimulus conditions with increasing distance from the human end of the continua (see **Figure 3C**). There was no significant difference between arousal ratings for M6 and human faces.

For the SAM valence ratings, there was a highly significant effect of morph position along the DHL, *F*(1.29,33.65) = 56.69, *p* < 0.001. Pre-planned contrasts showed a significant difference between avatar faces (*M* = 5.68; SD = 0.62) and M6 (*M* = 4.91; SD = 0.51), [*F*(1,26) = 59.75, *p* < 0.001], between M6 and the human faces (*M* = 4.55; SD = 0.68), [*F*(1,26) = 24.63, *p* < 0.001], and between avatar and human faces [*F*(1,26) = 36.31, *p* < 0.001]. These data show that the valence ratings increased negatively across the three stimulus conditions with increasing morph distance from the human end of the continua (see **Figure 3D**).

Please note that the levels ‘M0, M1, M2, M3’ and ‘M9, M10, M11, M12’ were selected for the preceding analyses to represent the avatar and human categories. But performing the same separate one-way RM-ANOVAs using just M0, M6, and M12 as the morph factor levels produced the identical pattern of results for LPP, EMG, SAM ratings for valence, and SAM ratings for arousal.

## Experiment 2: Ratings of familiarity

### Participants and Procedure

A sample of *N* = 30 participants aged between 20 and 30 years (15 female; *M* = 25.64 years; SD = 2.88) were examined. The laboratory, stimulus conditions, task requirements, and instructions in this study were the same as described for Task 2 (i.e., self-ratings of valence and arousal) of Experiment 1, except that participants were required to view and rate feelings of familiarity in response to stimuli on a 5-point Likert scale by pressing the appropriate response key as quickly and accurately as possible after stimulus onset. This task permitted the analysis of RTs for familiarity judgements. The rating scale ranged from very strange (1) to very familiar (5). A practice pre-test of five trials was applied, as described for the tasks of Experiment 1. Please note that the pilot study to determine facial attractiveness of continua endpoints, described in Section “Materials and Stimuli,” was the same as used for self-ratings of familiarity, except that a 5-point bipolar Likert rating scale ranging from very unattractive (1) to very attractive (5) was used.

## Results

### Familiarity Ratings

**FIGURE 5:**
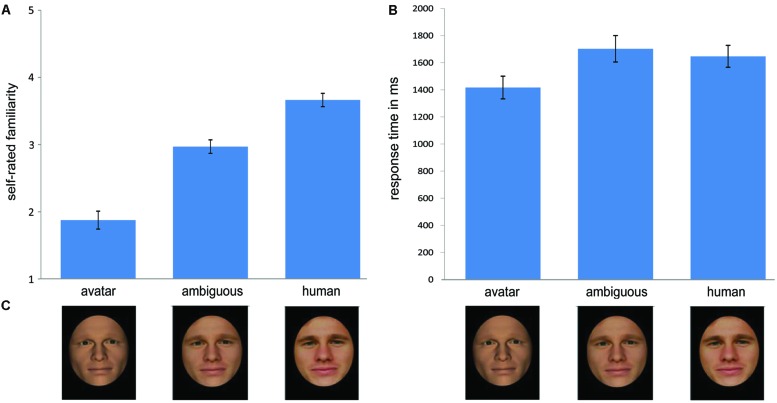
**Self-reported feelings of familiarity. (A)** Illustrates a general increase in familiarity ratings with increasing human likeness from the avatar to the human faces **(A)**. Reflecting the pattern of response times for categorization response times in the two-alternative forced choice categorization task, response latencies for ambiguous faces are longer compared with the avatar and human faces. The error bars indicate standard errors (*N* = 30). **(B)** Shows examples of morphs drawn from a continuum of the kind used to represent the DHL. The morph labeled M0 corresponds with the avatar endpoint of the continuum, M6 with greatest category ambiguity, and M12 with the human endpoint of the continuum. **(C)** Shows examples of stimuli used in this study.

A one-way RM-ANOVA with the factor *morph* position (three levels: M6, ‘M0, M1, M2, M3’ and ‘M9, M10, M11, M12’) and the dependent variables *familiarity* rating of each participant across the 20 continua revealed a highly significant effect of morph position, *F*(1.27,36.85) = 109.03, *p* = <0.001. Pre-planned contrasts showed a significant difference between the avatar morphs (*M* = 1.9; SD = 0.72) and M6 (*M* = 3.01; SD = 0.54), [*F*(1,29) = 134.42, *p* = <0.001] and between M6 and the human morphs (*M* = 3.68; SD = 0.54), *F*(1,29) = 48.58, *p* = <0.001, such that familiarity ratings increased negatively across the three stimulus conditions with increasing distance from the human end of the continua (see **Figure 5A**).

### RT of Familiarity Ratings

The same analysis, using instead RT for familiarity ratings also showed a highly significant effect of morph position, *F*(1.27,36.85) = 12.71, *p* = <0.001 (see **Figure 5B**). Reflecting the pattern of RT for categorization in Task 3 of Experiment 1, pre-planned contrasts showed a significant difference between RT at M6 (*M* = 1682; SD = 527) compared with the mean average for avatar *and* human morphs (*M* = 1482; SD = 376), *F*(1,26) = 20.41, *p* < 0.001, indicating that RT for familiarity judgments is significantly longer at M6 than for categorically unambiguous morphs. Pre-planned contrasts showed also that RT for familiarity ratings of avatars (*M* = 1400; SD = 453) was significantly faster than for human faces (*M* = 1627; SD = 437), *F*(1,29) = 11.9, *p* = 0.002.

## Discussion

The main prediction of the UVH is that observation of highly humanlike characters or objects that are difficult to distinguish from the human counterpart will elicit negative affect, whereas the affective experience of distinctly non-human and human characters or objects will be more positive in comparison. To test this, measures of valence and arousal (i.e., the primary orthogonal dimensions of affective experience) were used to compare the impact on affective state of categorically ambiguous faces with that of categorically unambiguous avatar and human faces; the physical morph distance of the avatar and human faces from the ambiguous faces was controlled for. To reflect Mori’s idea, we assumed that any evidence in support of an uncanny-like effect (i.e., enhanced negative affective experience for ambiguous faces) during passive viewing and during explicit affect evaluation would most likely be revealed by comparing the categorically most ambiguous stimuli with the most unambiguous stimuli. But the data from the LPP, EMG, and SAM-based measures of arousal and valence converge in showing no support for the notion that category ambiguity along the DHL is specifically associated with enhanced experience of negative affect. On the contrary, the LPP and SAM-based measures indicate a general increase in arousal and negative valence across the stimulus conditions (i.e., the human category, ambiguous category, avatar category) with increasing morph distance from the human end of the continua. The *ad hoc* familiarity ratings delivered the same picture, indicating that feelings of strangeness are not specifically associated with categorically ambiguous faces and generally increase with increasing morph distance from the human end of the continua.

These findings are consistent with the profile of subjective evaluations reported in other studies that have used comparable, well-controlled morph continua and *ad hoc* measures of shinwakan, such as pleasantness (e.g., Looser and Wheatley, 2010). This profile is characterized by a general asymmetry in affective experience, with increasingly negative evaluations of morphs with decreasing human likeness. But asymmetry along the DHL is not specific to affective processing. Asymmetries have also been reported in tasks of perceptual and category processing in other uncanny-related studies (Cheetham et al., 2011, 2013, 2014). These tasks have revealed greater decision certainty and shorter RT latencies in categorization judgments for avatar compared with human faces, extraction of different perceptual details from avatar compared with human faces during perceptual decision making, differential sensitivity of affect-related brain structures (e.g., amygdala, insula) to avatar compared with human faces during passive viewing, and enhanced discrimination sensitivity to perceptual differences in visual information between faces within the avatar compared with those within the human category. The data of the present study are therefore worth considering in the context of such asymmetries in perceptual and category processing.

The RT data in the categorization task (i.e., Task 3 of Experiment 1) show that explicit categorization judgments for avatar faces are faster than those for human faces. One interpretation of this finding is that different perceptual features are used for processing category information in novel avatar compared with “everyday” human faces, as indicated for implicit and explicit processing of perceptual and category information along the DHL in other studies (Cheetham et al., 2011, 2013, 2014). It has been suggested that this difference might relate to the use of novel and thus salient perceptual information as a readily identifiable feature of avatar faces (e.g., novel color, smoothed skin texture, or feature shape) to facilitate category processing (Cheetham et al., 2013). Assuming that novel information is easier to extract from the avatar faces compared with the corresponding category information from the human faces and that this information is preferentially used as diagnostic of avatar category membership, one might expect an *RT facilitation effect* such that RT latencies for category judgments of novel avatar faces are shorter compared with those of human faces (see Levin and Angelone, 2001). The present data are consistent with this idea (for an alternative explanation of RT facilitation, see Valentine, 1991).

While there was no requirement to categorize our stimuli during the other tasks (i.e., during passive observation, explicit evaluation of affect, and familiarity judgments), it is likely that participants did engage in implicit processing of the categories (for evidence of this during passive observation, see Cheetham et al., 2011; see also Castelli et al., 2004). If implicit processing of category did occur, a similar RT facilitation effect might be expected for our affective evaluations of avatar faces (please note that for experimental reasons, RT was only collected for familiarity judgments). This suggestion assumes that processing of category membership contributes to or influences in some way the processing of familiarity (for categorization effects and the evaluation of attractiveness, see Halberstadt and Winkielman, 2014). In fact, the data show that the latency of familiarity judgments is shorter for avatar than for human faces. The data show also that the latency of familiarity judgments is longest for the categorically most ambiguous faces. Familiarity judgments thus appear to be influenced by or interact with the processing and cognitive representation of the category structure of the continua (cf., e.g., Heekeren et al., 2008). Similar effects might apply for the psychophysiological and for the SAM-based measures of valence and arousal. The use of a different task design that allows clear interpretation of RT data of the SAM-based measures of valence and arousal might be used to examine this.

The preceding considerations hint at the possibility that there is a relationship of some kind between cognitive processing efficiency (as indicated in the preceding by RT), perceptual and category processing of the DHL, and our measures of affective experience of DHL stimuli. Further analysis of the familiarity ratings supports the idea of a relationship, revealing a highly significant correlation for avatars only, such that shorter RT for category judgments of avatars is associated with more negative familiarity ratings (i.e., greater strangeness), *r* = 0.575, *p* > 0.001. This finding is not consistent with the recent *inhibitory-devaluation hypothesis* that has been presented as a potential explanation for the uncanny valley (Ferrey et al., 2015). Ferrey et al.’s (2015) hypothesis posits that a stimulus that is subject to competing interpretations, such as when membership of a stimulus to one or other potential category is ambiguous, will be evaluated more negatively (for further details regarding the role of inhibitory cognition in this). Based on the use of RT to indicate decision difficulty due to competing interpretations of categorically ambiguous stimuli (see also Yamada et al., 2013), our data reveal no significant relationship between RT and familiarity ratings for categorically ambiguous faces and that the only significant relationship found (i.e., for avatar faces) shows longer RT for more positive ratings. The data of both experiments in Burleigh and Schoenherr’s (2015) recent study, based on ratings of eeriness, also lend no support for the inhibitory-devaluation hypothesis.

An alternative account of the uncanny effect that has attracted attention in uncanny research considers the influence of *processing fluency* on affective experience. According to the *Hedonic Fluency Model* (Winkielman et al., 2003), negative evaluations of novel or unfamiliar stimuli relate to subjective difficulty extracting diagnostic information for quick and efficient processing (see also Bradley et al., 1993; Bornstein and D’Agostino, 1994; Mendes et al., 2007). This proposal ties in well with the idea that negatively valenced experience along the DHL might be associated with category ambiguity. Yamada et al. (2013) follow the Hedonic Fluency Model and suggest on the basis of their data that lower processing fluency, as indicated in their study by categorization decision difficulty (i.e., longer RT) for categorically ambiguous stimuli of the DHL, is associated with enhanced negative judgments of likeability. In contrast, our data indicate that any change in arousal, valence and familiarity is not modulated by effects of category ambiguity and, specifically in relation to the correlative result between RT and the faces of the avatar category, do not favor the hedonic fluency account.

It might be argued that the correlative relationship between RT and familiarity ratings for avatars fits more closely with a different model of processing fluency, the *Fluency Amplification Model* (Albrecht and Carbon, 2014). Albrecht and Carbon show that stimuli with a comparatively neutral or negative valence at the outset are not liked any more under conditions of higher processing fluency than they are under conditions of lower processing fluency. They show also that higher processing fluency of negative stimuli can actually enhance negative evaluation. Our data are consistent with the possibility that higher processing fluency of avatar faces (i.e., shorter RT latencies) and enhanced negative evaluation might be related in this way. This possibility is reinforced by the data of a recent study showing that higher processing fluency, indicated by reduced difficulty in perceptual discrimination between highly similar faces along the DHL, correlates with negative affect evaluations of familiarity (i.e., enhanced feelings of strangeness; Cheetham et al., 2014); please note that this finding is entirely contrary to the effect predicted on the basis of the UVH.

The reported increase in negative valence and arousal for avatar compared with the human faces in the present study might simply relate to an innate predisposition to treat the unfamiliar with caution (Zajonc, 1998). The strength of caution diminishes as further exposure reveals that the unfamiliar is non-threatening (Lee, 2001). Correspondingly, the relatively more positive evaluations of valence for human faces might simply reflect the impact of repeated exposure to human category examplars. Previous social interaction and the often more positive affective tone of interaction with a particular in-group is thought to lead to automatic activation of more positive evaluations (Reis and Gable, 2003; Claypool et al., 2007; Garcia-Marques et al., 2010). This *mere-exposure* effect (Zajonc, 1968; Monahan et al., 2000; Zajonc, 2001) might underpin the general increase in pleasantness and liking ratings with increasing human likeness of faces in other studies (e.g., Looser and Wheatley, 2010; Cheetham et al., 2014; see Experiment 1 in Seyama and Nagayama, 2007), such that more humanlike faces (or their human-specifying perceptual features) are evaluated as more likeable (see, Moreland and Zajonc, 1982). A recent study investigated the idea that the frequency of exposure to the faces of fictive beasts modulates affective ratings of eeriness (Burleigh and Schoenherr, 2015), but the authors report only nearly significant effects. Given that these fictive faces were not manipulated in terms of human likeness, the potential impact of mere-exposure on affective ratings of highly humanlike faces is open to further consideration.

One possible consideration is that the mere-exposure effect is mediated by the history of normal social interaction and a tendency to individuate in-group but not out-group members (Ostrom et al., 1993). This *differential processing bias* might also apply when processing human and humanlike faces (see Cheetham et al., 2013). This bias means that human participants preferentially code other members of the human in-group (i.e., our human stimuli) by directing cognitive processing resources toward more in-depth processing of facial information to enable individuation (i.e., processing at the exemplar level). In contrast, the processing of out-group members (i.e., our highly humanlike avatar faces) might be biased toward facial information that enhances detection of faces at the category level; for this kind of out-group bias by other names, see the *other-race hypothesis* (Levin, 2000), *differential processing hypothesis* (Ostrom et al., 1993), and the *other-race effect* (Rhodes et al., 2009). More in-depth processing for individuation would be consistent with the longer RT latencies for categorization of our human category faces in Task 3 of Experiment 1, since longer latency suggests more time-consuming processing of finer perceptual details (Schyns and Murphy, 1994; Lamberts, 1998; Johansen and Palmeri, 2002). Assuming that longer RT of familiarity ratings for human faces also reflects more in-depth processing, the allocation of more attentional resources needed for this might be sufficient to strengthen any effects of mere-exposure on positive evaluations of faces (Huang and Hsieh, 2013). This explanation is consistent with the present data and it could be investigated further in relation to categorization performance. It is worth noting, however, that Cheetham et al. (2014) did already test the *differential processing bias* as a potential explanation for their finding of an asymmetry along the DHL in perceptual discrimination, that study showing enhanced perceptual discrimination of avatar faces compared with human faces. The data in that study did not support this explanation, and, in terms of perceptual discrimination, lend little support to Schoenherr and Burleigh’s (2015) suggestion that an out-group bias might also underpin certain social-cultural phenomena that resemble elements of the uncanny valley idea.

We applied a two-dimensional approach to examining affective experience by placing the focus in the main experiment on arousal and valence. The data show a consistent pattern in the relationship between lesser degrees of human likeness and greater arousal (i.e., in the LPP and self-rating SAM measures) and more negative valence (i.e., in the self-rating SAM measure). The combination of negative valence and increased arousal is understood as indicating negative affective experience (Lang et al., 2005). But the EMG measure of corrugator supercilii activity showed no effects along the DHL. A straightforward interpretation of this would be that human likeness along the DHL has no differential impact on the valence of actual affective state during passive viewing. But in keeping with Larsen et al. (2003), who report a strong relationship between corrugator supercilii activity and self-reported valence ratings, further analysis of the data of each participant across continua also shows a strong positive relationship between increasing corrugator activity and more negative valence ratings (*r* = 0.474, *p* = 0.006). However, this effect is specific for the human category, suggesting that the valence of actual feeling state (as indexed by the EMG measure) and the cognitive evaluation of feeling state (as indexed by SAM measure) strongly converge for human faces only. This convergence might reflect a close coupling of the representation and integration of affective and cognitive processing of actual feeling state and of the cognitive appraisal of that state in relation to external input (Schwarz and Clore, 1983, 2006) that might be acquired through repeated exposure and social interactive experience with human others. This human-specific effect might relate to the processing of facial mimicry acquired in normal social interaction with human others (Weyers et al., 2006). The comparatively weak effect for non-human faces might thus reflect an attenuated responsiveness to the neutral expression of non-human faces.

The data show a significantly greater (i.e., more positive-going) LPP for the avatar compared with the human category faces. It is possible that a larger number of trials (i.e., use of more continua) might have increased the chance of also finding a significant difference between these two face categories and the ambiguous faces. Huffmeijer et al. (2014) recommends 30 trials to capture the LPP, whereas Moran et al. (2013) demonstrate that the LPP is stable and can be quantified with as few as 12 trials. Based on previous studies (Cuthbert et al., 2000; Yen et al., 2010; Herbert et al., 2013; Moran et al., 2013), we considered 20 continua to be adequate to test Mori’s ideas. Considered in the context of the general decrease in LPP amplitude with increasing human likeness of the morphs, it is likely that any such difference between the ambiguous and the unambiguous faces would still reflect this general decrease and not produce an uncanny-like effect. But further investigations could examine this possibility by using more trials per morph level. It should be noted also that while the LPP is modulated by arousing (negatively and positively valenced) stimuli (e.g., Schupp et al., 2000; Leite et al., 2012; Weinberg et al., 2012), valence can also modulate the LPP (e.g., Cuthbert et al., 2000; Delplanque et al., 2006). The valence effect is less consistently reported (Olofsson et al., 2008), but we cannot exclude the possibility that effects of both arousal and valence are reflected in the LPP data. This would not change the findings of the present study, as arousal and valence are primary dimensions of affective experience.

The use of psychophysiological indices of affect introduces additional sources of data to the investigation of Mori’s ideas. Together with the SAM-based ratings, this approach delivers a more complete picture of the underlying components of affective experience. In the present study, this picture includes direct and objective measures of psychophysiological reactions to the DHL stimuli and indirect and subjective measures based on the cognitive evaluation of the stimuli and of the consciously detected psychophysiological reactions that these evoke. The appeal of psychophysiological indices is that their measurement is conducted continuously and in real time, meaning that physiological events can be detected as they unfold over time at a temporal resolution on a millisecond scale. For example, the LPP develops at around 300–400 ms after stimulus onset, peaks at around 700 ms, and lasts for up to 6 s in total (Cuthbert et al., 2000). Similarly, EMG can provide effective measurement of minuscule and rapid changes, including changes that escape detection by the naked eye (e.g., Cacioppo et al., 1986; Dimberg, 1990; Weyers et al., 2006; Gomez et al., 2009; Mauss and Robinson, 2009; van Boxtel, 2010; Likowski et al., 2011; Wu et al., 2012). In view of the poor construct definition of the affective dimension described in the UVH, that some individuals find it difficult to conceptualize and quantify their emotional experiences (Mauss and Robinson, 2009), and that (*ad hoc*) self-reports might not well capture the constructs that they are intended to measure (Ho et al., 2008), further use of psychophysiological measures might contribute to a clearer characterization of the relationship between stimuli defined along the DHL and affect.

To characterize this relationship and to enable comparison between studies, the way in which the DHL is represented is an important consideration. One approach to its representation is to use morph continua (e.g., Seyama and Nagayama, 2007; for an alternative approach, see, e.g., Ho et al., 2008; Tinwell, 2009). The use of morphing permits close examination of the relationship between affect and experimentally controlled fine-grained differences in humanlike appearance. The general increase in positive affect toward the human end of the DHL indicated by our LLP, SAM arousal, and SAM valence (and by our familiarity) measures is reflected in the data of other studies based on subjective ratings of affect and on comparable nonhuman–human morph continua (e.g., Experiment 1 in Seyama and Nagayama, 2007; Looser and Wheatley, 2010; Cheetham et al., 2014). But there are findings inconsistent with the present data. The most similar study is that of Yamada et al. (2013). They examined the relation between explicit ratings of likeability and category ambiguity along a morph continuum generated from the face of the cartoon character Charlie Brown and a human face. While they reported negative affect specifically in relation to category ambiguity, they indicate also that morphing disparities in the alignment of facial features may have influenced subjective ratings. Inspection of their stimuli suggests that these disparities particularly affected the categorically most ambiguous morphs by creating the appearance of a facial scar across the forehead of the morphed faces. This kind of morphing artifact is likely to have had a systematic effect on subjective ratings as it is related to the morph distance from the continua endpoints (see Cheetham and Jancke, 2013).

MacDorman and Ishiguro (2006) also used non-human and human morphs to represent the DHL, reporting an uncanny valley-like negative peak in affective ratings. But their use of more than one juxtaposed continuum to represent the DHL combined with non-equivalent increments of physical change between the morphs of the DHL (see also Hanson et al., 2005) appears to have created the stimulus conditions needed to generate an uncanny-like effect in the profile of subjective responses. It should be noted that the experimental manipulation of facial features along morph continua for the explicit purpose of evoking uncanny-like effects has been applied in other experiments (see Experiments 2 and 3 in Seyama and Nagayama, 2007). Other studies have used experimentally controlled morph continua and examined affect along similar dimensions of facial human likeness. However, these have used computer-generated rather than natural human faces to represent the human end of the human likeness dimension (MacDorman et al., 2009a). Considered in terms of the UVH, the human endpoints in these studies are in effect exemplars of the non-human category. Use of computer-generated faces to represent the human faces is not unusual in face research (e.g., Todorov et al., 2009). But there are, as discussed in the preceding, differences in perceptual and category processing between natural human and computer-generated human-like faces. Importantly, perceptual and category processing of human category and similar computer-generated, non-human category faces can correlate differently with measures of affect, as shown for perceptual discrimination (Cheetham et al., 2014) and in the present study. In terms of the UVH, this makes comparison between such studies and those that use natural human faces difficult. Burleigh et al. (2013) and Burleigh and Schoenherr (2015) have also investigated the UVH using experimentally controlled morph continua, but their face morphs were not manipulated in terms of human likeness.

The present study focused on affective experience in terms of the dimensions arousal and valence. Affective experience can be conceptualized in other ways (see e.g., Davidson, 1993; Panksepp, 1998; Lindquist et al., 2012), and examination of arousal and valence is not new in emotion research (e.g., Lane et al., 1999; Robinson and Compton, 2006; Lewis et al., 2007; Demanet et al., 2011). But this two-dimensional approach is different than that taken in uncanny research to date (e.g., Ho et al., 2008; Tinwell, 2009; Ho and MacDorman, 2010). For example, Ho et al. (2008) focus on defining specific emotions, such as fear, with which to characterize uncanny experience. But it is worth noting that Ho et al. (2008) see parallels between their own findings (using robotic stimuli), arousal and valence, and Russell’s *circumplex model of affect* (Russell, 1980). This model understands affective states, such as fear, as arising from neurophysiological systems that relate to arousal and valence (for a detailed review, see Posner et al., 2005). It is conceivable that measures of arousal and valence might explain a significant amount of the variance in and provide further insight into the affective constructs (e.g., likeability, feelings of familiarity, fear, disgust) typically used to investigate the uncanny effect (for overviews, see Ferguson and Bargh, 2003; Posner et al., 2005; Cunningham and Zelazo, 2007; Schwarz, 2007). We note, however, that psychophysiological measures do not replace measures of self-reported feelings, because self-reports and measures of psychophysiological reactivity and behavior are all relevant to the description of an emotional response (Mandler et al., 1961; Lang, 1989). Whether the present findings might generalize to fine-grained manipulations of the DHL based on computer-generated stimuli using different software to generate avatars (with manipulations of different perceptual information), female face stimuli, emotionally expressive faces, and dynamic stimuli (see Chaminade et al., 2007) is open to further investigation.

## Conflict of Interest Statement

The authors declare that the research was conducted in the absence of any commercial or financial relationships that could be construed as a potential conflict of interest.

## Footnotes

1 http://www.abrosoft.com

2 http://www.smithmicro.com
