# Supplementary material for: Arousal, valence, and the uncanny valley: psychophysiological and self-report findings
Source: Front Psychol. 2015 Jul 15;6:981. doi: 10.3389/fpsyg.2015.00981 (PMC4502535; doi:10.3389/fpsyg.2015.00981)
Supplement: Supplementary file 1 [file Image_1.PDF]

Arousal, valence, and the uncanny valley: Psychophysiological and self-report findings.

Marcus Cheetham, Lingdan Wu, Paul Pauli, Lutz Jancke

Supplemental Information 1

Twenty morph continua were selected for the present study on the basis of two preceding pilot studies (N = 63). These used a *two-alternative forced choice classification task* identical to the task described in Experiments 1 (see also Supplemental Information 2) to determine the category structure of continua based on avatar versus human category responses. The criteria for selection was that the aggregated means across continua for the morph positions M0, M1, M2, and M3 (i.e. avatar end of DHL), for the morph positions M9, M10, M11, and M12 (i.e. human end of DHL), and for M6 (i.e. most closely associated with greatest ambiguity) would be consistent across continua and show a clear degree of categorisation certainty for avatar and human exemplars (see Supplemental Figure 1).

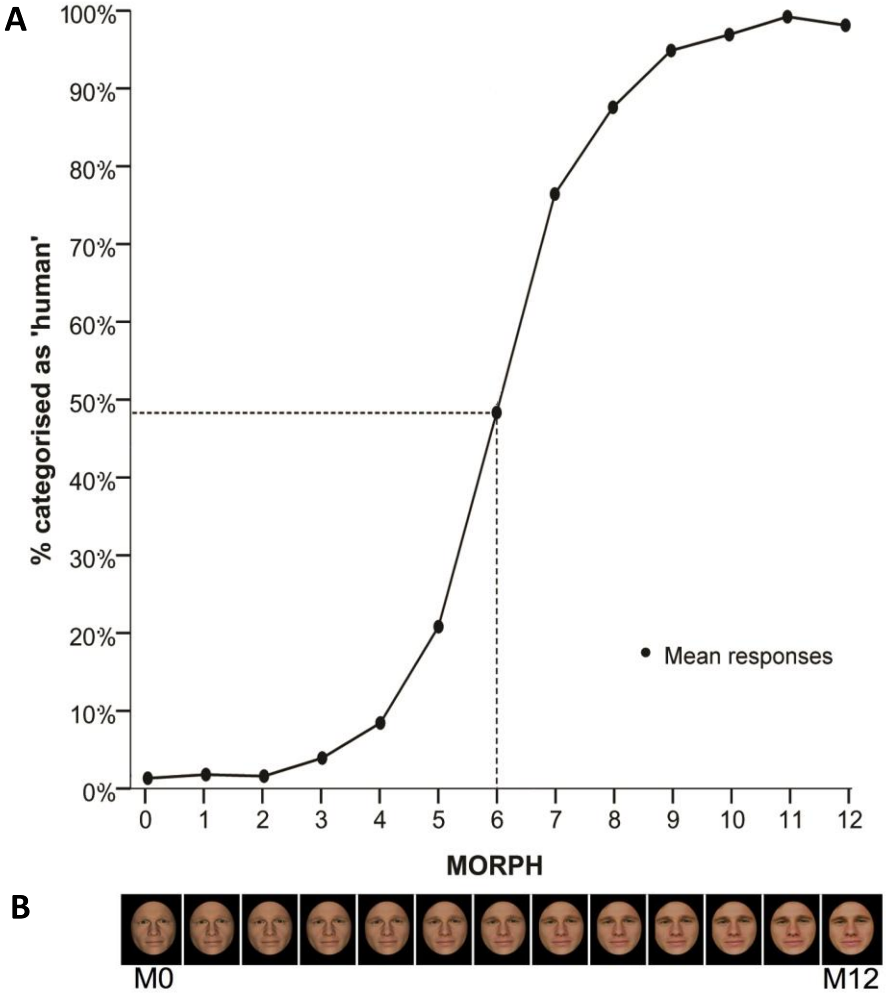

Supplemental Figure 1. Panel **A** shows mean aggregate category decisions in the *two-alternative forced choice classification task* for all morphs in the pilot studies (N=63). The mean aggregated decision responses (shown with continuous interpolation line) are illustrated in terms of the percentage of 'human' categorisation decisions. The figure shows that morph 6 is almost perfectly aligned with the point of maximum uncertainty of 50% in categorization judgments (dashed line) and that morphs 'M0, M1, M2, M3' and 'M9, M10, M11, and M12' are unambiguously judged to be avatar and human category faces, respectively. Panel **B** shows an

example of a morph continuum of the kind used in the present study. M0 refers to the avatar endpoint and M12 to the human endpoint of the continuum.

The resulting pilot data (considered in terms of percentage of categorization judgments for the human category) showed that across continua the morph positions M0, M1, M2, and M3 were unambiguously assigned to the avatar category (M = 0.02; SD = 0.02), M6 was most closely associated with greatest ambiguity (i.e. 50%) in categorization judgments (M = 0.48; SD = 0.11), and M9, M10, M11, and M12 were unambiguously assigned to the human category (M = 0.98; SD = 0.02) (see Supplemental Figure 1). It should be noted that a much less conservative criterion is regularly used in research of categorization decision making to indicate category (e.g., 66% as in Ectoff and Magee, 1992; Beale and Keil, 1995). To test for consistency across continua, a one-way repeated measures of analysis of variance with the factor 'morph position' (9 levels: M0, M1, M2, M3, M6, M9, M10, M11, M12) was conducted on the dependent variable categorization *response* of each participant for the 20 continua. The 9 selected morphs represent the morph positions used in the analyses of data in experiments 1 and 2, that is, M0, M1, M2, and M3 representing the unambiguous avatar category, M6 for greatest category ambiguity, and M9, M10, M11, and M12 representing the unambiguous human category. There were no significant differences between the 20 continua, $F(19, 152) = 1.44$, $p = 0.12$. Data for response times were not analysed as these were not relevant for the tasks in the main experiment.

Supplemental Information 2

After completion of the other tasks in Experiment 1 (i.e. passive viewing and SAM-based self-ratings) a two-alternative forced choice classification task was conducted to verify the response profile in terms of categorisation ambiguity.

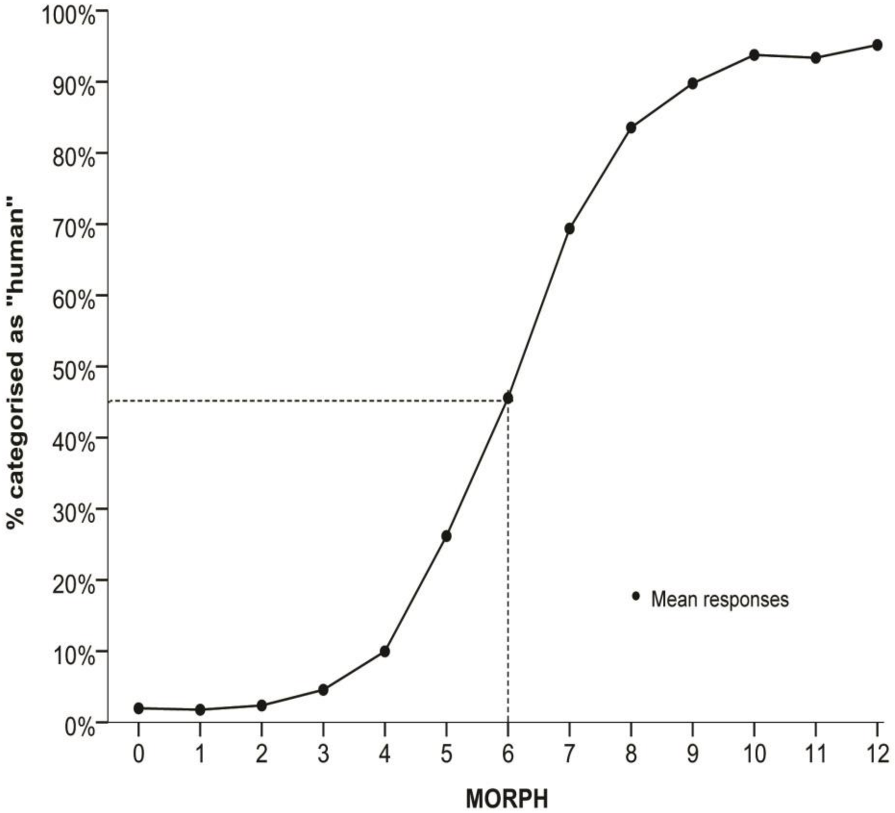

Supplemental Figure 2. Mean aggregate category decisions in the *two-alternative forced choice classification task* for all morphs in Experiment 1. The mean aggregated decision responses (shown with continuous interpolation line) are illustrated in terms of the percentage of 'human' categorisation decisions. The figure

shows that morph 6 is most closely aligned with the point of maximum uncertainty of 50% in categorization judgments (dashed line) and that morphs 'M0, M1, M2, M3' and 'M9, M10, M11, and M12' are unambiguously judged to be avatar and human category faces, respectively.

References

Ectoff, N. L., & Magee, J. J. (1992). Categorical perception of facial expressions. *Cognition, 44*, 227–240.

Beale, J. M., & Keil, F. C. (1995). Categorical effects in the perception of faces. *Cognition, 57*, 217–219.
